# Association between calcitonin receptor *Alu*I gene polymorphism and bone mineral density: A meta-analysis

**DOI:** 10.3892/etm.2014.2083

**Published:** 2014-11-20

**Authors:** QI XIONG, LINGLI XIN, LIHAI ZHANG, ZHI MAO, PEIFU TANG

**Affiliations:** 1 Department of Orthopedics, General Hospital of Chinese PLA, Beijing 100853, P.R. China; 2 Department of Obstetrics and Gynecology, The Second Artillery General Hospital of Chinese PLA, Beijing 100088, P.R. China

**Keywords:** calcitonin receptor gene, bone mineral density, polymorphism

## Abstract

The association between calcitonin receptor (CTR) *Alu*I gene polymorphism and bone mineral density (BMD) remains unclear. In order to elucidate this association, a meta-analysis was performed to provide a comprehensive assessment of the studies carried out to date. PubMed, the Cochrane Library, Web of Science and the China National Knowledge Infrastructure database were searched to identify eligible studies. The data were extracted independently by two authors using a standard form, the studies were meta-analyzed and disagreements were resolved through discussion. Fifteen eligible studies involving 3,093 females and 654 males were included for analysis. Overall, the male subjects with the CC genotype had non-statistically different lumbar spine and femoral neck BMD compared to subjects with the CT/TT and CT genotypes. The BMD of female subjects with the CC genotype was similar to that of patients with the CT or CT/TT genotypes. In Chinese male subjects, those with the CC genotype had almost the same BMD as those with the CT and CT/TT genotypes. The results also demonstrated that Chinese female subjects with the CC genotype had similar BMD at the lumbar spine and femoral neck to subjects with the CT and CT/TT genotypes. Furthermore, Southern Chinese subjects with CC genotypes did not have a different BMD at the lumbar spine and femoral neck compared to patients with CT and CT/TT genotypes. Notably, Northern Chinese subjects with the CC genotype had a higher BMD at the lumbar spine compared to subjects with CT/TT genotypes and a lower BMD at the femoral neck compared to subjects with CT/TT genotypes. Among Northern Chinese females, those with CC genotypes also had a higher BMD at the lumbar spine compared to those with CT/TT genotypes, while no difference was observed in the BMD of the lumbar spine and femoral neck between patients with CC and CT genotypes. In Southern Chinese females, no significant difference was found in the BMD at the lumbar spine and femoral neck between those with CC and those with CT or CT/TT genotypes. In conclusion, the *Alu*I gene polymorphism may have an association with BMD in Northern Chinese subjects and the CC genotype may have a protective effect on spine BMD; however, the CC genotype may be a risk factor for low femoral neck BMD in Northern Chinese subjects. Further studies are required to fully investigate the potential association between *Alu*I gene polymorphism and BMD.

## Introduction

Osteoporosis is a systemic disorder affecting the skeletal system and is characterized by a reduced bone mass and micro-architectural deterioration of bone tissue with a consequent increase in bone fragility and susceptibility to fracture (1). Bone mineral density (BMD) is commonly used as a skeletal phenotype in evaluating osteoporosis. The World Health Organization defines osteoporosis as a BMD value of ≥2.5 standard deviations below the young-adult mean measured by dual-energy X-ray absorptiometry (DXA) (2). The pathophysiology of osteoporosis is complex and involves numerous endogenous (genetic and hormonal) and environmental factors. Twin and family studies have shown that genetic influences account for 50–80% of the inter-individual variability of BMD in young adults (3–5). Various candidate genes have been implicated in the genetic basis of osteoporosis, including hormones and their receptors, cytokines and bone-matrix proteins. Polymorphisms in the genes encoding the calcitonin receptor (CTR), estrogen receptor (ESR) and vitamin D receptor (VDR) have been studied previously and the results show that these receptors are positively or negatively associated with biomarkers of bone turnover, BMD and the incidence of osteoporotic fracture (6–8). Genome-wide association studies and meta-analysis have confirmed the association between BMD and ESR or VDR (9–11). To the best of our knowledge, there have been no genome-wide association studies or meta-analyses to assess the association between *Alu*I gene polymorphism and BMD.

Calcitonin, a 3.4-kDa polypeptide hormone secreted by thyroid gland parafollicular cells, is an important hormone regulating calcium metabolism and bone turnover through the CTR. The CTR, which is expressed in osteoclasts and osteoclast precursor cells, activates one of the members of the G-protein-coupled receptor family. By doing this, it regulates bone metabolism and maintains the calcium balance between bone resorption and formation (12,13).

In 1997, Nakamura *et al* (14) described an *Alu*I CTR polymorphism in the Japanese population, which was characterized by a single nucleotide difference at position 1,377 of human CTR cDNA, expressing either proline (CCG) or leucine (CTG) as the amino acid at position 463. Single nucleotide polymorphisms are used as a tool for mapping the disease gene. Using this technique, Masi *et al* (15) found an association between the *Alu*I CTR gene C/T polymorphism and BMD in Italian postmenopausal females. Furthermore, Tsai *et al* (16) reported that an *Alu*I CTR gene polymorphism was associated with a reduced BMD, and predisposed postmenopausal females to osteoporosis; however, other studies reported contrasting results. Charopoulos *et al* (17) reported that *Alu*I polymorphism was not associated with BMD in Greek males, as no significant difference was observed in the BMD between CTR genotypes. Xu *et al* (18) also found that CTR gene polymorphism had no evident effect on Xinjiang Han and Uygur postmenopausal patients with osteoporosis, and the authors suggested that CTR gene polymorphism was not involved in the low bone mass. Consequently, no conclusion about the association between *Alu*I polymorphism and BMD could be drawn.

As the small sizes and different ethnicities of individual studies may be responsible for the contrasting results, a large-scale study with more subjects is required. Meta-analysis is an effective tool that is frequently used to compensate for the limitations of individual studies by pooling all published data together to obtain sufficient statistical power to detect potential effects of small to moderate sizes of samples associated with these polymorphisms. In order to explore the effect of *Alu*I polymorphism on BMD, a meta-analysis was therefore performed in the present study to provide a more comprehensive assessment of the association between *Alu*I CTR gene polymorphisms and BMD in an elderly population, particularly in China.

## Materials and methods

### Ethics statement

The present meta-analysis was conducted according to the Preferred Reporting Items for Systematic Reviews and Meta-analyses guidance with minor modifications appropriate for this study (19), and did not require ethics board approval.

### Literature searching

A literature search for eligible studies published prior to March 31, 2014 was conducted in the following electronic databases: PubMed, Web of Science, Cochrane Library and the China National Knowledge Infrastructure. The following combined keywords and MeSH terms were used: ‘calcitonin receptor’ [All Fields] or ‘CTR’ [All Fields] or ‘AluI’ [All Fields] or ‘rs1801197’ [All Fields], and ‘genes’ [MeSH Terms] or ‘gene’ [All Fields], and ‘polymorphism, genetic’ [MeSH Terms] or ‘polymorphism’ [All Fields] or ‘genetic polymorphism’ [All Fields] and ‘bone density’ [MeSH Terms] or ‘bone density’ [All Fields] or ‘bone mineral density’ [All Fields] or ‘BMD’ [All Fields]. Studies written in English and Chinese focusing on middle-aged or older subjects were included. The reference lists of reviews and retrieved articles were manually screened by two independent authors to identify additional potential studies.

### Inclusion and exclusion criteria

To be included in the analysis, the candidate studies had to meet the following criteria: i) Genotyping was performed with validated molecular methods and the possible genotypes were CC, CT or TT for *Alu*I; ii) lumbar spine and femoral neck BMD was measured by DXA; and iii) measurements of BMD at the lumbar spine and/or femoral neck were used to calculate the mean difference and their corresponding 95% confidence intervals (95% CI). Studies were excluded for the following reasons: i) Duplicate publication; and ii) subjects younger than 18 years old. If a research team reported similar data in different studies, the study reporting the largest number of subjects was included. In addition, when raw and adjusted BMD values were available, adjusted BMD values were used. When the complete information required for quantitative synthesis was unavailable, the relevant authors were contacted to obtain the necessary information.

### Data extraction

For eligible studies, information was extracted on authors, publication year, country and region, age, the number of subjects recruited, genotypes and the BMD of the lumber spine and femoral neck in each genotype. All data were extracted independently by two authors using a standard form, and minor discrepancies were resolved through discussion by the authors.

### Statistical analysis

A statistical test (Cochran’s Q statistic) of heterogeneity was used to evaluate any potential inter-study heterogeneity: P<0.05 indicated significant inter-study heterogeneity. Heterogeneity was also assessed through the I^2^ test, with I^2^>50 indicating significant heterogeneity. When no heterogeneity was found, a fixed-effect model was used to estimate the pooled mean differences and their corresponding 95% CIs; otherwise, a random-effect model was applied. The following comparisons were evaluated: Patients with the CC genotype versus patients with the CT/TT or the CT genotype. Subgroup analyses were conducted by region and gender. Egger’s regression test was performed to assess the publication bias. All statistical tests were two-tailed. P<0.05 indicated a statistically significant difference. All the analyses were performed with Comprehensive Meta Analysis V2 software package (Biostat, Inc., Englewood, NJ, USA).

## Results

### Characteristics of the eligible studies

**Figure 1:**
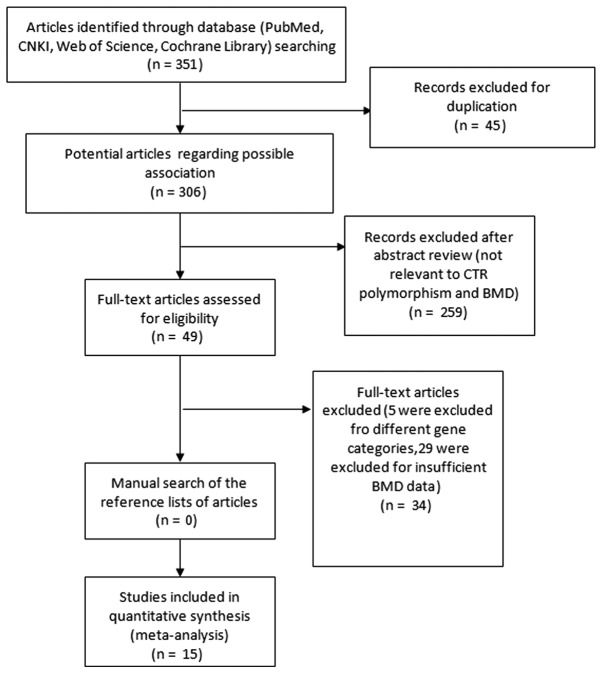
Detailed information on the selection of eligible studies. CTR, calcitonin receptor; BMD, bone mineral density; CNKI, China National Knowledge Infrastructure.

Fig. 1 shows detailed information on how the studies were selected. There were 15 eligible studies with 3,093 females and 654 males (16,20–33). Table I shows further detailed information on the eligible studies. Two studies recruited subjects in Italy (20,21), one in Japan (30) and 12 in China. Three studies recruited only male subjects (21,24,26), one study recruited both male and female subjects (31) and 11 studies recruited only female subjects. The majority of subjects recruited were postmenopausal females. BMD measurements in all 15 studies were performed by DXA, although with different instruments. The BMD values of both the lumbar spine and femoral neck were measured in 14 of the studies, with one study measuring only the lumbar spine BMD (30). In three of the 15 eligible studies, the BMD value was adjusted for age and weight (16,20,21). Three of the studies had combined CT and TT data (indicated as CT/TT), without raw CT or TT data (22,24,30). Genotyping was carried out in a consistent manner across studies using validated polymerase chain reaction methods. As it is not possible to introduce substantial bias for BMD values and genotype, the studies did not specify whether measurements were blinded.

### Meta-analyses for AluI polymorphism effects on lumbar spine BMD

**Figure 2:**
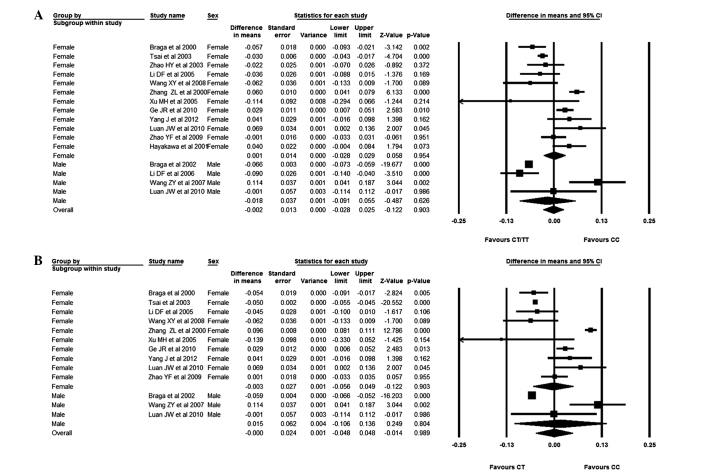
Forest plot of the meta-analysis in all subjects. (A) CC versus the CT/TT genotype; (B) CC versus the CT genotype. Subgroup analysis was based on gender. The difference in bone mineral density for various *Alu*I genotypes contrasted at the lumbar spine. The random-effects model was used. CI, confidence interval.

As subjects with the TT genotype are rare compared with those with either CC or CT genotypes, comparisons were only made between patients with CC and CT genotypes or those with CC and CT/TT genotypes. In male subjects, the weighted mean difference (WMD) for the CC versus the CT/TT genotypes was −0.018 (95% CI, −0.091–0.055), and the WMD for the CC versus the CT genotypes was 0.015 (95% CI, −0.106–0.136). Considering the female subjects, the BMD difference for subjects with the CC genotype versus those with the CT/TT or CT genotypes was −0.001 (95% CI, −0.028–0.029) and −0.003 (95% CI, −0.056–0.049), respectively. It was observed that patients with the CC genotype had a slightly lower BMD than patients with the CT or CT/TT genotype, although no significant association between *Alu*I and BMD could be found (Fig. 2).

**Figure 3:**
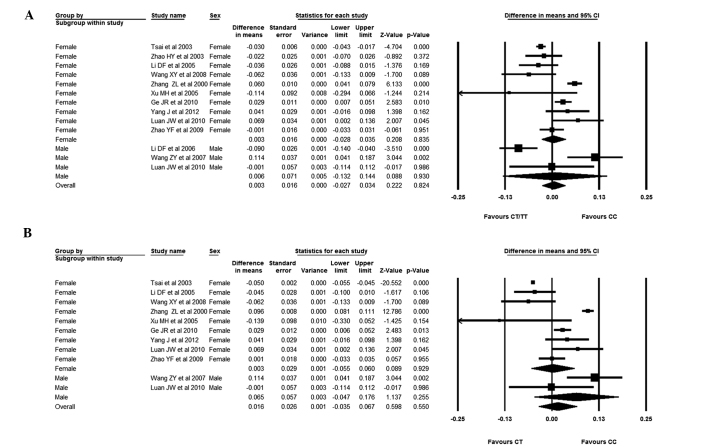
Forest plot of the meta-analysis in Chinese subjects. (A) CC versus the CT/TT genotypes; (B) CC versus the CT genotypes. Subgroup analysis was based on gender. Difference in bone mineral density for various *Alu*I genotypes contrasted at the lumbar spine. The random-effects model was used. CI, confidence interval.

To clarify whether *Alu*I polymorphisms had an effect on lumbar spine BMD in a Chinese cohort, the studies recruiting subjects from countries other than China (20,21,30) were excluded. In Chinese male and female subjects, those with the CC genotype had a higher BMD than those with the CT genotype. The WMD for patients with the CC genotype versus those with the CT genotype was 0.065 (95% CI, −0.047–0.176) in male subjects and 0.003 (95% CI, −0.055–0.060) in female subjects. The BMD difference between patients with the CC genotype and those with the CT/TT genotype was monitored and, similarly, a higher BMD in male and female subjects with the CC genotype was observed. The WMD for patients with the CC genotype versus those with the CT/TT genotype was 0.006 (95% CI, −0.132–0.144) and 0.003 (95% CI, −0.028–0.035) in male and females, respectively (Fig. 3).

**Figure 4:**
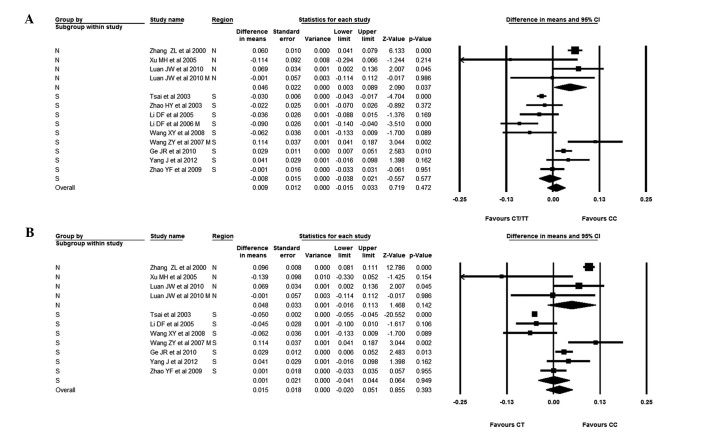
Forest plot of the meta-analysis in Chinese subjects. (A) CC versus the CT/TT genotypes; (B) CC versus the CT genotypes. Subgroup analysis was based on region. Difference in bone mineral density for various *Alu*I genotypes contrasted at the lumbar spine. The random-effects model was used. N, Northern; S, Southern; CI, confidence interval.

With regard to subgroup analysis for subjects from Southern and Northern China, it was found that subjects with the CC genotype from Southern China had a slightly lower BMD than subjects with the CT genotype; the WMD for the CC versus the CT genotype was 0.001 (95% CI, −0.041–0.044). In subjects from Northern China, it was observed that those with the CC genotype had a higher BMD than those with the CT genotype, with a BMD difference of 0.048 (95% CI, −0.016–0.113). When comparing patients from Northern China with the CC genotype versus those with the CT/TT genotypes, patients with the CC genotype were found to have a significantly higher BMD. The BMD difference was 0.046 (95% CI, 0.003–0.089) (Fig. 4).

**Figure 5:**
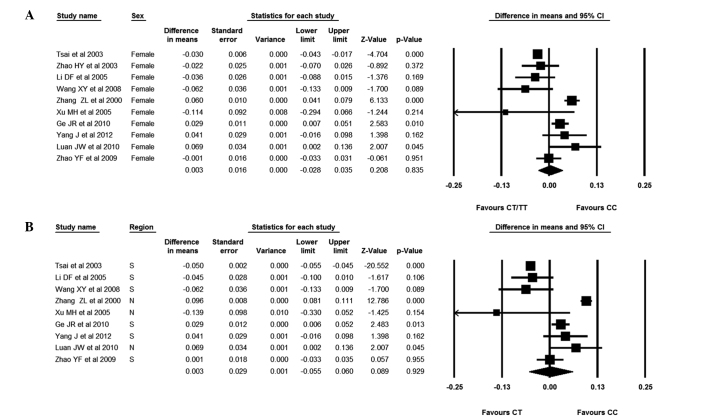
Forest plot of the meta-analysis in Chinese female subjects. (A) CC versus the CT/TT genotype; (B) CC versus the CT genotype. Difference in bone mineral density for various *Alu*I genotypes contrasted at the lumbar spine. The random-effects model was used. N, Northern; S, Southern; CI, confidence interval.

**Figure 6:**
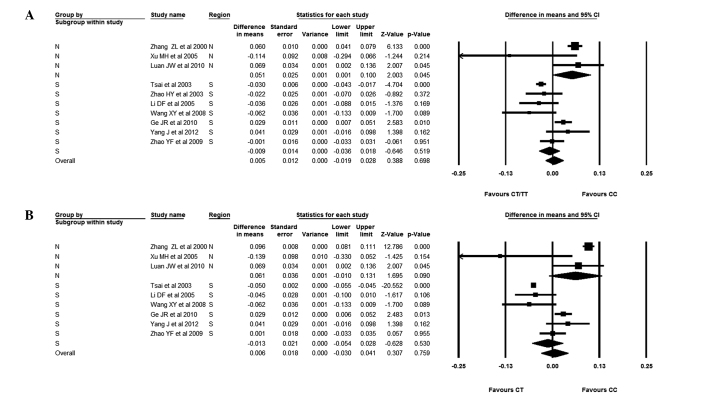
Forest plot of the meta-analysis in Chinese female subjects. (A) CC versus the CT/TT genotypes; (B) CC versus the CT genotypes. Subgroup analysis was based on region. Difference in bone mineral density for various *Alu*I genotypes contrasted at the lumbar spine. The random-effects model was used. N, Northern; S, Southern; CI, confidence interval.

The focus was subsequently changed to the association between *Alu*I polymorphisms and BMD in Chinese females. It was observed that patients with the CC genotype had a slightly higher BMD than those with the CT/TT genotype; the WMD was 0.003 (95% CI, −0.028–0.035). There was, however, no statistical difference between subjects with the CC and CT/TT genotypes. The females were also divided into Southern and Northern groups. In the females from Southern China, those with the CC genotype had a lower BMD than those with the CT/TT genotype; the WMD was −0.009 (95% CI, −0.036–0.018). It was evident, however, that females from Northern China with the CC genotype had a higher BMD than those with the CT/TT genotype; the WMD was 0.051 (95% CI, 0.001–0.100). Finally, the studies without BMD values for patients with the CT genotype were excluded. Chinese females with the CC genotype were compared with those with the CT genotype; the WMD was 0.003 (95% CI, −0.055–0.060). It was observed that individuals with the CC genotype had a higher BMD than those subjects with the CT genotype, although the difference was not significant. It was also found that Northern female subjects with the CC genotype had slightly, but not significantly, higher BMDs than those with the CT genotype; the WMD was 0.061 (95% CI, −0.010–0.131). In Southern female subjects, those with the CC genotype had a lower BMD than those with the CT genotype; the WMD was −0.013 (95% CI, −0.054–0.028) (Figs. 5 and 6).

### Meta-analyses for AluI polymorphism effects on femoral neck BMD

**Figure 7:**
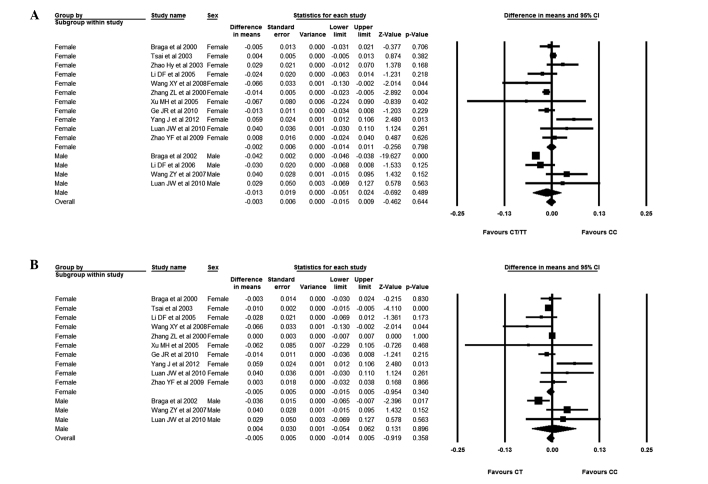
Forest plot of the meta-analysis in all subjects. (A) CC versus the CT/TT genotype; (B) CC versus the CT genotype. Subgroup analysis was based on gender. Difference in bone mineral density for various *Alu*I genotypes contrasted at the femoral neck. The random-effects model was used. CI, confidence interval.

In male subjects, the mean BMD of the femoral neck was lower in subjects with the CC genotype, although there was no significant difference between those with the CC and the CT/TT genotypes. The WMD was −0.013 (95% CI, −0.051–0.024). Similarly, the mean BMD in female subjects with the CC genotype was lower than that in subjects with the CT/TT genotype; the WMD was −0.002 (95% CI, −0.014–0.011), showing no statistical difference between subjects with the CC and CT/TT genotypes. Subsequently, as for the lumbar spine evaluation, the mean femoral neck BMD of subjects with the CC genotype was compared with that of subjects with the CT genotype. It was observed that male subjects with the CC genotype had a higher BMD compared with subjects with the CT genotype; the WMD was 0.004 (95% CI, −0.054–0.062). By contrast, female subjects with the CC genotype had a lower BMD, with the WMD being −0.005 (95% CI, −0.015–0.005). There was, however, no statistical difference between those with the CC genotype and those with the CT genotype in both male and female subjects (Fig. 7).

**Figure 8:**
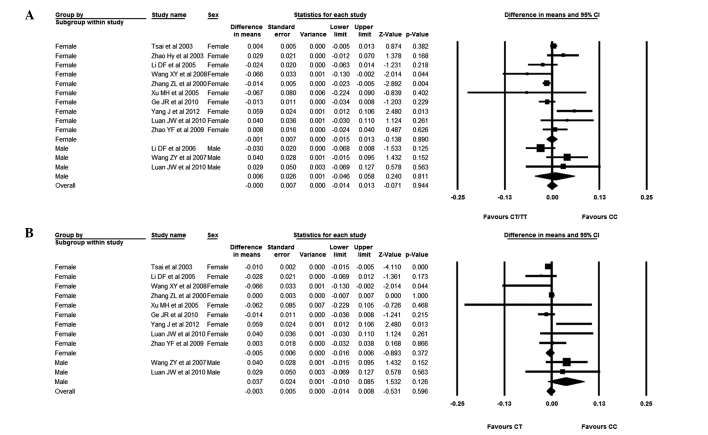
Forest plot of the meta-analysis in Chinese subjects. (A) CC versus the CT/TT genotype; (B) CC versus the CT genotype. Subgroup analysis was based on gender. Difference in bone mineral density for various *Alu*I genotypes contrasted at the femoral neck. The random-effects model was used. CI, confidence interval.

The Chinese subjects were then considered to confirm whether there was any association between *Alu*I polymorphism and BMD. Patients with the CC genotype were compared with patients with the CT/TT genotype. The results showed that male patients with the CC genotype had a higher BMD than those with the CT/TT genotype, while female patients with the CC genotype had a lower BMD than those with the CT/TT genotype; the WMDs were 0.006 (−0.046–0.058) and −0.001 (−0.015–0.013), respectively. Patients with the CC genotype were then compared with those with the CT genotype. There were just two studies that recruited Chinese male subjects (24,26) and these showed that patients with the CC genotype had a slightly higher BMD than those with the CT genotype; the BMD difference was 0.037 (95% CI, −0.010–0.085). In Chinese female subjects, however, those with the CC genotype had a lower BMD than subjects with the CT genotype; the WMD was −0.005 (95% CI, −0.016–0.006). No significant BMD difference was observed between Chinese subjects with the CC and CT or CT/TT genotypes (Fig. 8).

**Figure 9:**
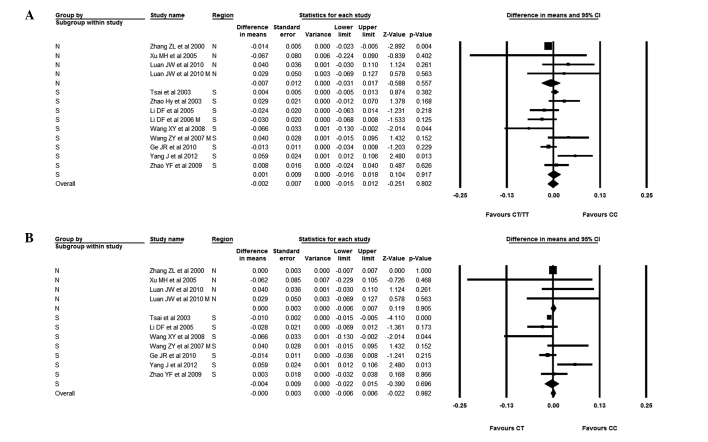
Forest plot of the meta-analysis in Chinese subjects. (A) CC versus the CT/TT genotypes; (B) CC versus the CT genotypes. Subgroup analysis was based on region. Difference in bone mineral density for various *Alu*I genotypes contrasted at the femoral neck. The random-effects model was used. N, Northern; S, Southern; CI, confidence interval.

The Chinese subjects were then divided into Southern and Northern groups; the BMD difference was −0.013 (95% CI, −0.022--0.003). In Southern subjects, the BMD of those with the CC genotype was not significantly different from that of subjects with the CT/TT genotype; the BMD difference was 0.001 (95% CI, −0.016–0.018). The BMD was similar in Northern Chinese subjects when considering those with the CC and CT genotypes; the BMD difference was (95% CI, −0.006–0.007). In Southern Chinese subjects, however, those with the CC genotype had a slightly lower BMD than those with the CT genotype. The difference was −0.004 (95% CI, −0.022–0.015) (Fig. 9).

**Figure 10:**
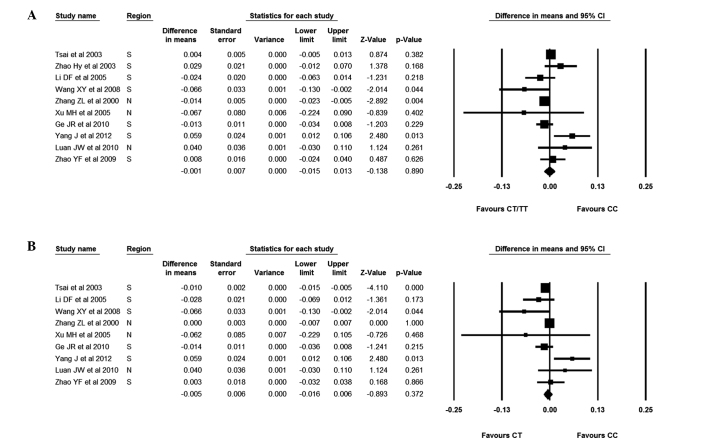
Forest plot of the meta-analysis in Chinese female subjects. (A) CC versus the CT/TT genotypes; (B) CC versus the CT genotype. Difference in bone mineral density for various *Alu*I genotypes contrasted at the lumbar spine. The random-effects model was used. N, Northern; S, Southern; CI, confidence interval.

**Figure 11:**
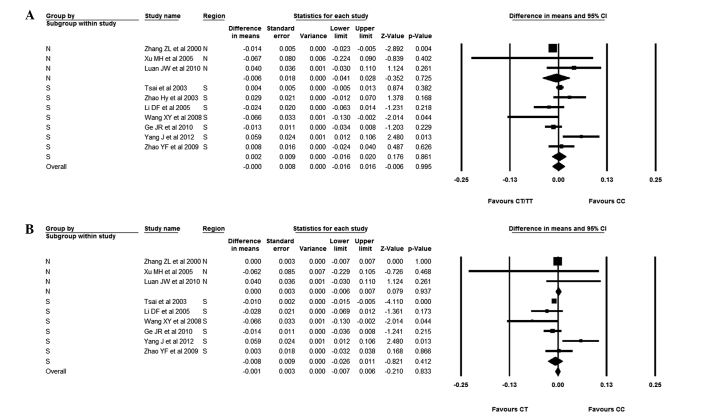
Forest plot of the meta-analysis in Chinese female subjects. (A) CC versus the CT/TT genoypes; (B) CC versus the CT genotypes. Subgroup analysis was based on region. Difference in bone mineral density for various *Alu*I genotypes contrasted at the lumbar spine. The random-effects model was used. N, Northern; S, Southern; CI, confidence interval.

Attention was finally focused on the effect of polymorphism on femoral neck BMD in Chinese female subjects. No significant difference was found between subjects with the CC genotype and subjects with the CT/TT genotype; the BMD difference was −0.001 (95%CI, −0.015–0.013). This group was then divided into Chinese female subjects from either the South or the North of China. It was observed that patients with the CC genotype had statistically lower BMDs than those with the CT/TT genotype but only in subjects from Northern China; the BMD difference was −0.013 (95% CI, −0.023--0.004). No significant difference was found, between subjects with the CC genotype and those with the CT/TT genotype in Southern Chinese females, although subjects with the CC genotype had a higher BMD than those with the CT/TT genotype [BMD difference, 0.002 (95% CI, −0.016–0.020)]. Chinese female subjects with the CC genotype and those with the CT genotype were also compared. The results showed that subjects with the CC genotype had a lower BMD than those with the CT genotype; the BMD difference was −0.005 (95% CI, −0.016–0.006). The Chinese female subjects with the CC and CT genotypes were then divided into Northern and Southern subgroups. In the Northern female subjects, those with the CC genotype had a similar BMD to those with the CT genotype; the BMD difference was 0.000 (95% CI, −0.006–0.007). By contrast, those with the CC genotype had a slightly lower BMD than subjects with the CT genotype in Southern China; the WMD was −0.008 (95% CI, −0.026–0.011) (Figs. 10 and 11).

### Publication bias assessment

Publication bias was assessed by Egger’s regression test for all comparisons. Publication bias of subjects with the CC genotype versus those with the CT/TT genotypes at the lumbar spine and femoral neck was found (P<0.1). In the other comparisons no significant publication bias was observed (P>0.1 for comparisons of the CC and CT genotypes and for the CC and CT/TT genotypes in Chinese subjects).

## Discussion

This meta-analysis was conducted as findings on the association between *Alu*I polymorphism and BMD are incongruous. The present study pooled the data on the association between the *Alu*I polymorphism and BMD at the lumbar spine and femoral neck in 3,747 subjects. As the frequency of the TT genotype was rare in the Chinese population, the study compared patients with the CC genotype with patients with the CT or CT/TT genotypes. The results demonstrated that, in Asia and Europe, subjects with the CC genotype had a slightly lower BMD than those with the CT/TT genotype and a slightly higher BMD than those with the CT genotype; however, no difference in BMD was found between male subjects with the CC genotype and those with the CT/TT or CT genotypes, and this was consistent with previous studies (17,18). At the femoral neck the results were similar, with no difference found between patients with the CC genotype and those with the CT or CT/TT genotypes. In combination, the results suggested that *Alu*I polymorphism had no effect on lumbar spine and femoral neck BMD in male subjects, although the CC genotype in males may have a protective effect at the femoral neck but be a risk factor in the lumbar spine. When considering the female subjects, those with the CC genotype had a lower BMD at the lumbar spine and femoral neck than those with the CT or CT/TT genotypes. The results suggested that the CC genotype served as a risk factor in female subjects. Despite this, a statistical difference was not observed between individuals with the CC genotype and those with the CT or CT/TT genotypes. Similarly, the implication is that *Alu*I polymorphism has no effect on BMD.

The subjects of 12 eligible studies were Chinese in this meta-analysis; therefore, particular attention was focused on Chinese subjects to explore the association between *Alu*I polymorphism and BMD. At the lumbar spine, the results showed that subjects with the CC genotype had a higher BMD than subjects with the CT and CT/TT genotypes, although the difference was not significant. These results suggested that the CC genotype may have a protective effect on the lumbar spine BMD; however, no significant difference was found between patients with the CC genotype and those with the CT or CT/TT genotypes. At the femoral neck, the results were at variance with those at the lumbar spine. In Chinese female subjects, those with the CC genotype had a lower BMD than those with the CT/TT genotype, yet the difference was not significant; this indicated that the CC genotype had a converse effect on the femoral neck to that on the lumbar spine. No association was therefore found between *Alu*I polymorphism and BMD.

Since the Southern and Northern Chinese populations share a different diet, behavior and environment, subgroup analysis of Chinese subjects was carried out in accordance with the region. Notably, in Northern subjects, a significantly lower femoral neck BMD was observed in subjects with the CC genotype versus that in subjects with the CT/TT genotype, while those with the CC genotype had a statistically higher lumbar spine BMD compared with patients with the CT/TT genotype. These results demonstrated that *Alu*I polymorphism had an association with BMD in Northern Chinese patients, with the CC genotype having a protective effect on the lumbar spine whilst serving as a risk factor at the femoral neck. The results of the present study were partly consistent with the results in Korea reported by Lee *et al* (34). Lee *et al* also found that subjects with the CC genotype had a higher BMD at the lumbar spine; however, the same study also reported that patients with the CC genotype had a higher BMD at the femoral neck, which was in contrast to the results revealed here. Furthermore, Bandrés *et al* (7) reported a statistically significant association between the CTR gene polymorphism and BMD in Spanish females. A common factor among these findings is that they were all from subjects from Northern regions; however, the results themselves showed significant variation. The explanations for this phenomenon remain to be elucidated, and the mechanism underlying the association requires clarification.

As females are more susceptible to osteoporosis than males, the association between *Alu*I polymorphism and BMD was specifically investigated in Chinese female subjects. The results showed that there was no difference in the BMD of the lumbar spine and femoral neck between subjects with the CC genotype and those with the CT/TT or CT genotypes. The Chinese female subjects were then divided into Southern and Northern groups. The results suggested that, in the Northern subjects, those with the CC genotype had a statistically higher lumbar spine BMD than those with the CT/TT genotype, and subjects with the CC genotype had a trend of high femoral neck BMD, although this was not significant. No difference, however, was identified in Southern subjects, similar to subjects from China as a whole. In combination, it may be suggested that *Alu*I polymorphism had an association with the BMD of the lumbar spine in Northern Chinese females.

This meta-analysis had a number of limitations. As shown in previous studies (22,35,36), the distribution of allelic frequency is different in Asia and Europe, and the majority of the individuals included in the present study were Chinese; therefore, data from different ethnicities is required to identify the exact association of *Alu*I polymorphism with BMD. In addition, only published studies were included so publication bias cannot be absolutely excluded, although no significant publication bias was observed by Egger’s regression test in the majority of the comparisons. Furthermore, the small number of subjects with the TT genotype led to comparisons only of patients with the CC and CT or combined CT/TT genotypes, which reduced the statistical power of the study, and insufficient data from male subjects made the analysis of the association between *Alu*I polymorphism and BMD in male subjects problematic. Finally, the interaction between other risk genes and the CTR gene may also contribute to the pathology of a reduced BMD, which could not be tested due to insufficient data.

In conclusion, the present study suggested that the *Alu*I gene polymorphism may have an association with BMD in Northern Chinese subjects, and the CC genotype may have a protective effect on BMD at the lumbar spine; however, the CC genotype may also serve as a risk factor for low femoral neck BMD in Northern Chinese subjects. Further studies with larger sample sizes and different ethnicities and genders are required to clarify the association.

## Figures and Tables

**Table I:** Detailed information of the 15 eligible studies.

First author, year (ref.)	Genotype	Gender	Region	n	Age (years)	LS BMD	FN BMD
Braga, 2002 (21)	CC	M	Italy	45	52.64±2.45	0.914±0.026	0.759±0.017
	CT	M	Italy	111	57.41±1.56	0.973±0.018	0.795±0.010
	TT	M	Italy	97	55.55±1.71	0.988±0.018	0.807±0.011
	CT/TT	M	Italy	208	56.54±1.87	0.980±0.019	0.801±0.012
Braga, 2000 (20)	CC	F	Italy	77	61.09±12.44	0.752±0.169	0.644±0.110
	CT	F	Italy	296	64.35±11.34	0.806±0.144	0.647±0.109
	TT	F	Italy	342	63.42±11.13	0.812±0.151	0.651±0.111
	CT/TT	F	Italy	638	63.85±11.23	0.809±0.148	0.649±0.110
Tsai, 2003 (16)	CC	F	Taiwan	123	54.17±6.25	0.99±0.01	0.81±0.01
	CT	F	Taiwan	37	54.14±4.44	1.04±0.02	0.82±0.02
	TT	F	Taiwan	4	55.25±6.34	0.83±0.07	0.68±0.05
	CT/TT	F	Taiwan	41	54.25±4.57	1.020±0.069	0.806±0.048
Zhao, 2003 (22)	CC	F	CHN Shanghai	321	48.42±16.47	1.050±0.177	0.878±0.152
	CT/TT	F	CHN Shanghai	62	46.35±16.46	1.072±0.182	0.849±0.150
Li, 2005 (23)	CC	F	CHN Guangzhou	194	60±8.3	0.6145±0.14	0.6468±0.11
	CT	F	CHN Guangzhou	33	63±7.8	0.6601±0.19	0.6750±0.11
	TT	F	CHN Guangzhou	4	63±4.3	0.5790±0.09	0.6387±0.09
	CT/TT	F	CHN Guangzhou	37	63±7.46	0.6513±0.18	0.6711±0.11
Li, 2006 (24)	CC	M	CHN Guangzhou	205	72±6	0.65±0.13	0.64±0.11
	CT	M	CHN Guangzhou				
	TT	M	CHN Guangzhou				
	CT/TT	M	CHN Guangzhou	42	70±5	0.74±0.23	0.67±0.14
Wang, 2008 (25)	CC	F	CHN Anhui	230	61.8±6.5	0.773±0.112	0.720±0.102
	CT	F	CHN Anhui	10	63.6±7.5	0.835±0.134	0.786±0.086
	TT	F	CHN Anhui	0			
	CT/TT	F	CHN Anhui	10	63.6±7.5	0.835±0.134	0.786±0.086
Zhang, 2002 (33)	CC	F	CHN Beijing	118	Postmenopause	0.903±0.015	0.734±0.010
	CT	F	CHN Beijing	7	Postmenopause	0.807±0.057	0.734±0.010
	TT	F	CHN Beijing	2	Postmenopause	0.971±0.108	0.799±0.075
	CT/TT	F	CHN Beijing	9	Postmenopause	0.843±0.096	0.748±0.040
Wang, 2007 (26)	CC	M	CHN Shenzhen	47	>70	0.908±0.115	0.668±0.086
	CT	M	CHN Shenzhen	12	>70	0.794±0.119	0.628±0.088
	TT	M	CHN Shenzhen	0	>70		
	CT/TT	M	CHN Shenzhen	12	>70	0.794±0.119	0.628±0.088
Xu, 2005 (27)	CC	F	CHN Hebei	52	53.2±11.8	1.021±0.253	0.785±0.220
	CT	F	CHN Hebei	7		1.160±0.115	0.847±0.127
	TT	F	CHN Hebei	1		0.961±0	0.885±0
	CT/TT	F	CHN Hebei	8		1.135±0.128	0.852±0.118
Ge, 2010 (28)	CC	F	CHN Fuzhou	422		0.759±0.125	0.807±0.119
	CT	F	CHN Fuzhou	152		0.766±0.119	0.821±0.120
	TT	F	CHN Fuzhou	17		0.765±0.122	0.809±0.105
	CT/TT	F	CHN Fuzhou	169	Postmenopause	0.766±0.119	0.820±0.118
Yang, 2012 (29)	CC	F	CHN Shanghai	102	Postmenopause	0.968±0.129	0.744±0.105
	CT	F	CHN Shanghai	25	Postmenopause	0.927±0.141	0.685±0.113
	TT	F	CHN Shanghai	0	Postmenopause		
	CT/TT	F	CHN Shanghai	25	Postmenopause	0.927±0.141	0.685±0.113
Hayakawa, 2001 (30)	CC	F	JPN	113	Premenopause	1.16±0.10	
	CT	F	JPN		Premenopause		
	TT	F	JPN		Premenopause		
	CT/TT	F	JPN	27	Premenopause	1.12±0.12	
Luan, 2010 (31)	CC	F	CHN Shandong	171	62±8.9	1.049±0.16	0.910±0.17
	CT	F	CHN Shandong	24	62±7.8	0.980±0.14	0.870±0.10
	TT	F	CHN Shandong	0			
	CT/TT	F	CHN Shandong	24	62±7.8	0.980±0.14	0.870±0.10
	CC	M	CHN Shandong	88	63±8.9	1.104±0.15	0.902±0.13
	CT	M	CHN Shandong	7	58±5.0	1.105±0.07	0.873±0.09
	TT	M	CHN Shandong	0			
	CT/TT	M	CHN Shandong	7	58±5.0	1.105±0.07	0.873±0.09
Zhao, 2009 (32)	CC	F	CHN Guangzhou	89	Postmenopause	0.742±0.083	0.682±0.084
	CT	F	CHN Guangzhou	26	Postmenopause	0.741±0.062	0.679±0.064
	TT	F	CHN Guangzhou	5	Postmenopause	0.752±0.058	0.647±0.033
	CT/TT	F	CHN Guangzhou	31	Postmenopause	0.743±0.061	0.674±0.061

M, male; F, female; CHN, China mainland; JPN, Japan; LS, lumbar spine; FN, femoral neck; BMD, bone mineral density (g/cm^2^); ref., reference number.
